# Identification the Cellular Senescence Associated lncRNA LINC01579 in Gastric Cancer

**DOI:** 10.1111/jcmm.70360

**Published:** 2025-01-24

**Authors:** Jiayong He, Ziyi Fu, Boya Zou, Xuetao Lei, Linhan Lei, Qingbin Yang, Guoxin Li

**Affiliations:** ^1^ Department of General Surgery, Nanfang Hospital Southern Medical University, Guangdong Provincial Engineering Technology Research Center of Minimally Invasive Surgery Guangzhou People's Republic of China; ^2^ Guangdong Provincial Key Laboratory of Precision Medicine for Gastrointestinal Tumor Guangzhou People's Republic of China; ^3^ The First School of Clinical Medicine Southern Medical University Guangzhou People's Republic of China; ^4^ Department of Dermatology Seoul National University College of Medicine Seoul Republic of Korea; ^5^ Laboratory of Cutaneous Aging Research, Biomedical Research Institute Seoul National University Hospital Seoul Republic of Korea; ^6^ Institute of Human‐Environment Interface Biology Seoul National University Seoul Republic of Korea; ^7^ Cancer Center of Beijing Tsinghua Changgung Hospital, School of Clinical Medicine,Tsinghua Medicine Tsinghua University Beijing People's Republic of China

**Keywords:** cellular senescence, fibroblast, gastric cancer, prognosis, tumour microenvironment

## Abstract

Cellular senescence is a key promoter of tumorigenesis and malignant progression. This study aimed to develop a predictive model for assessing cellular senescence in gastric cancer (GC) outcomes. We identified senescence‐related genes and lncRNAs from 375 stomach adenocarcinoma (STAD) patients and established a prognostic senescence score using multivariate Cox regression, validated in testing, TCGA‐STAD and the combined TCGA‐COAD and READ cohorts. The model's predictive efficacy was evaluated across clinical subgroups, tumour microenvironments and immune cell infiltration. A total of 116 senescence‐related lncRNAs were filtered, and patients were clustered into two senescent subtypes. The lncRNA signature identified LINC01579 as an independent prognostic factor for GC. Low‐risk groups showed prolonged overall survival, increased immune cell infiltration and reduced mutation load. Downregulation of LINC01579 using antisense oligonucleotides (ASOs) on normal human fibroblasts decreased cellular proliferation and migration in GC. Collectively, this study established and validated a promising prognostic model connecting senescence‐related lncRNAs and clinical outcome in GC and provided potential senescence‐related biomarkers for GC prognosis prediction.

## 1 Introduction

Gastric cancer (GC) represents a prominent global malignancy, ranking as the fifth most prevalent cancer and the third leading cause of mortality worldwide. Notably, GC has emerged as the primary or secondary cause of death in individuals below the age of 70 across numerous countries, as evidenced by the GLOBOCAN 2020 report [1]. This growing burden of cancer incidence and mortality can be attributed to the escalating prevalence of ageing populations. The incidence of GC is profoundly influenced by this demographic shift, with a median age of diagnosis typically occurring at 70 years [2].

Furthermore, a mounting body of empirical data underscores the inextricable link between ageing and cellular senescence, which concomitantly promotes the progression of cancer towards malignancy [3, 4]. Cellular senescence characterises an enduring cell cycle arrest that restricts the proliferative lifespan of cells, manifesting in response to external stressors [3, 5, 6]. In addition to impeding cell proliferation, the senescence program orchestrates alterations in cellular morphology and metabolism [5]. Notably, the senescence process induces the activation of a distinct phenotype termed senescence‐associated secretory phenotype (SASP) [3, 5, 7]. SASP orchestrates the release of bioactive molecules, including cytokines, growth factors, chemokines and proteases [7]. It is widely acknowledged as a potent driver of chronic diseases and cancer, intimately associated with DNA damage responses [8, 9], which are recognised as contributing factors to senescence, as well as dysregulated RNA processing [9, 10]. Notably, investigations have revealed that within the context of cancer, the ageing tumour microenvironment (TME) engenders reprogramming of stromal fibroblasts, the predominant constituents of the stroma, extracellular matrix and immune infiltration, thereby precipitating adverse cancer progression [11, 12, 13].

Long non‐coding RNAs (lncRNAs) are non‐coding RNAs with a length of more than 200 nucleotides. LncRNAs lack the ability to encode proteins, yet they play significant roles in the regulation of gene expression, encompassing transcription, mRNA stability, translation and protein stability [14, 15]. These molecules are recognised as crucial regulators of cellular proliferation and differentiation, exerting a profound influence on cellular senescence programs and various diseases, particularly cancer [16]. In addition to their involvement in regulating cellular activities, emerging evidence suggests that lncRNAs are part of the repertoire of regulatory RNAs that impact the SASP at multiple levels [11, 17, 18]. Nonetheless, the precise functions of cellular senescence‐related lncRNAs in cancer remain unclear.

**FIGURE 1:**
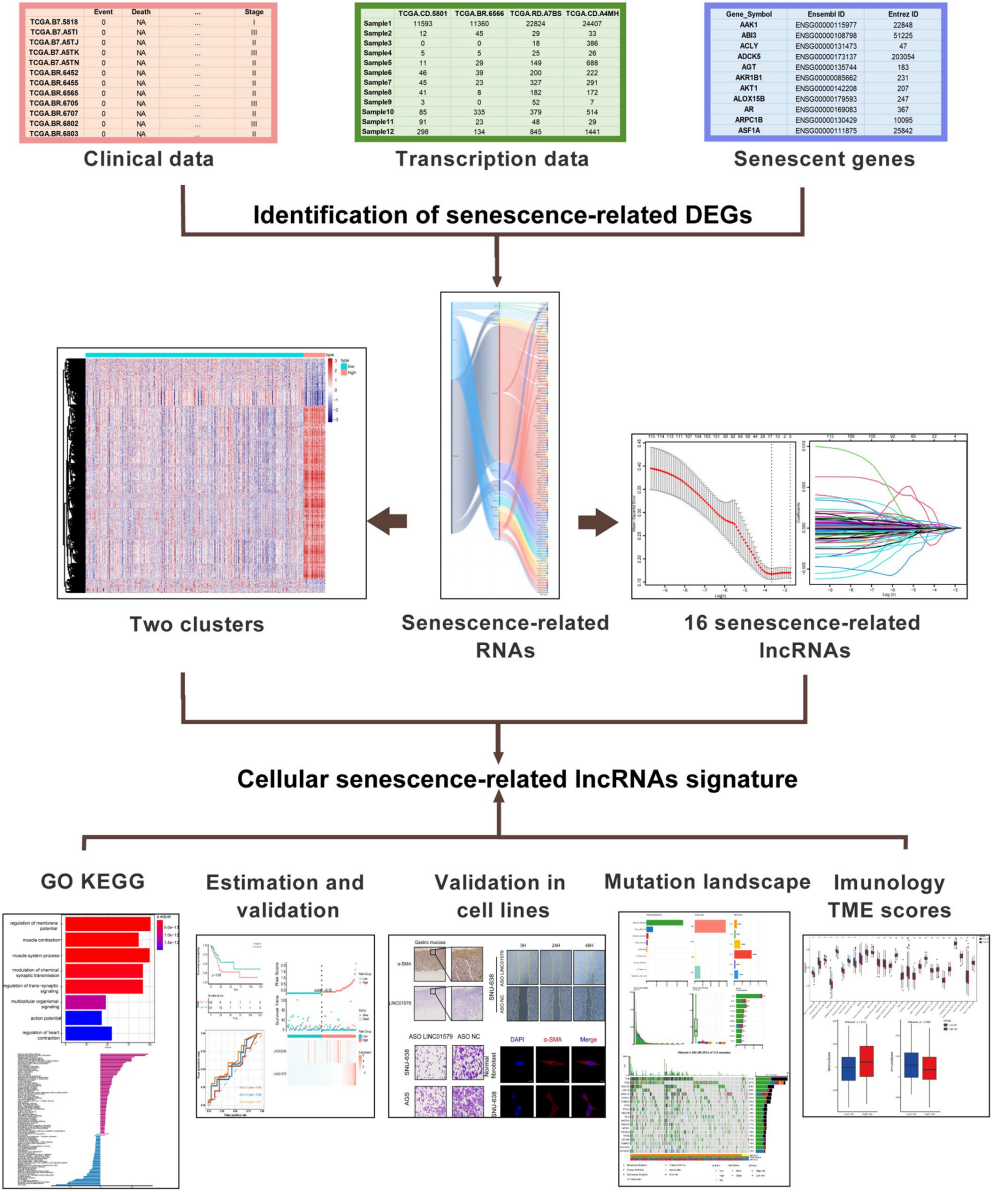
Workflow of this study.

In our study, a meticulous analysis was conducted to systematically evaluate the associations between cellular senescence‐related lncRNAs and prognosis in GC. Additionally, a senescence scoring system was developed and validated, utilising cellular senescence‐related mRNAs and lncRNAs sourced from the TCGA database. The primary objective of this scoring system was to predict overall survival (OS) and therapeutic prognosis for individual GC patients. Furthermore, in vitro experiments were carried out to investigate the impact of LINC01579 on the malignant progression of GC. The overall study design is shown in Figure 1 after referring the related bioinformatics studies [19, 20].

## 2 Materials and Methods

### 2.1 Data Acquisition

Clinical information and transcriptional profiles of patients with stomach adenocarcinoma (STAD), Colon adenocarcinoma (COAD) and rectum adenocarcinoma (READ) were obtained from The Cancer Genome Atlas (TCGA, https://portal.gdc.cancer.gov). The clinical information, RNA and lncRNA expression of 375 STAD patients were included after filtering. Human genes associated with cell senescence were downloaded from CellAge [21] (https://genomics.senescence.info/cells/).

### 2.2 Identification of Cellular Senescence‐Related lncRNAs

Differentially expressed genes (DEGs) were sorted out from the RNA expression of 375 STAD patients after filtering the information of normal tissues by package edgeR. DEGs and the gene list from CellAge were intersected to define the cellular senescence related RNAs. Correlations between senescence related RNAs and lncRNA expressed in TCGA‐STAD cohort were calculated using Spearman analysis. Genes with correlation score higher than 0.5 were defined as senescence‐related lncRNAs.

### 2.3 Construction of a Senescence Related lncRNA Signature for Outcome Prediction

Unsupervised hierarchical clustering analysis was conducted on 375 STAD samples of the TCGA set after filtering the information of normal tissues, using the set of 116 senescence‐related lncRNAs. All 375 samples were clustered into two groups, group A is the control group, while group B is the senescence relevant group. In order to explore the potential functions of the senescence‐related lncRNAs, we performed functional enrichment analysis to find significantly enriched Gene Ontology terms and Kyoto Encyclopedia of Genes and Genomes (KEGG) pathway, by clusterProfiler software in R‐ version 4.1.2. After filtering the information of normal tissues and the absence of clinical information, a multi‐gene signature with 16 senescence related lncRNAs was constructed on 325 STAD samples by LASSO regression, based on lamda. min and using package ‘survival’ and ‘glmnet’ [22, 23]. Multivariate Cox regression was used to sort out the senescence related lncRNA signature for outcome prediction. Then, risk score of each STAD patient was calculated based on the signature, using the formula as follows:

Senescence score $=\sum_{i=n}^{1}\text{coef}\left(\text{lncRNAi}\right)\times \text{expr}\left(\text{lncRNAi}\right).$

The formula calculates the prognostic risk score for STAD patients. lncRNAi represents the sorted‐out prognostic lncRNA and expr (lncRNAi) represents the expression level of lncRNAi for the patient. Scores of Coef (lncRNAi) were obtained from the regression of coefficient of multivariate Cox analysis, representing the contribution of lncRNAi to senescence score. Therefore, patients were divided into high‐risk group and low‐risk group by the median senescence score.

### 2.4 Evaluation and Verification of Senescence Score

Firstly, we drew the ROC curves of the whole set. Then, we divided the patients into two groups randomly by R, one as training set, the other as the testing set. We also obtained and combined the information of TCGA‐COAD and READ cohorts as an external testing set. Based on the senescence score, we also verified the efficacy of training set, testing set, and the combined TCGA‐COAD and READ cohorts respectively by ROC curves.

Moreover, we compared the OS respectively in the whole set, the training set, the testing set, and the combined TCGA‐COAD and READ cohorts between the high‐risk group and the low‐risk group, by log‐rank test. According to the clinical status of STAD patients, we also divided the patients into different clusters by the stage and age, thus compared the differences of patients in high‐risk and low‐risk group in each cluster.

The somatic mutation data were acquired from The Cancer Genome Atlas (TCGA, https://portal.gdc.cancer.gov). By the functions of maftools R package, the mutation landscape was then depicted. Data about the molecular subtypes of TCGA cohorts were sorted out as groups: the group A and B by unsupervised hierarchical clustering analysis.

### 2.5 Assessment of the Tumour Microenvironment and Immune Cell Infiltration

We assessed the proportions of components in the TME by the ESTIMATE algorithm of the ‘estimate’ package, respectively obtained the immune score, stromal score and the estimate score. Higher scores represent the greater proportion of the corresponding component in the TME.

### 2.6 Acquisition of Normal Fibroblasts (NFs)

The NFs were isolated from normal gastric tissue after Collagenase (Sigma, USA) and DNase (Sigma, USA) treatment. All the regents and supplies used in study were listed in Table S1.

### 2.7 Cell Culture, Transfection and Chemotherapeutics Treatment

Human AGS, SNU638 GC cell lines and NFs were cultured in RPMI 1640 with L‐Glutamine (Vivacell, Shanghai, China) containing 10% foetal bovine serum (Vivacell, Shanghai, China), maintained in cell incubator at 37°C and 5% CO₂. Antisense oligonucleotide (ASO) against LINC01579 (Tsingke, Beijing, China) was added with lipofectamine 3000 (Invitrogen, Carlsbad, CA, USA) in order to specifically suppress LINC01579 level in NFs. After transfection, chemotherapeutics treatment was utilised to induce cellular senescence. DNA topoisomerase II inhibitor Doxorubicin hydrochloride (Doxo) (MedChemExpress, New Jersey, USA) was used to induce cellular senescence in NFs, culturing for 48 h.

### 2.8 Immunofluorescence

NFs were washed by PBS, subsequently, 4% paraformaldehyde was added per dish for cells fixation at RT. Then, NFs were incubated with 0.2% Triton‐X‐100 (Solarbio, Beijing, China) for 5 min and were blocked by ready‐to‐use normal goat serum (Boster Biological Technology, China) for 1 h at RT. After that, primary antibodies against α‐SMA (Proteintech, USA) at 4°C overnight. Anti‐rabbit IgG (Cell Signaling Technology, USA) was utilised as secondary antibody to incubate cells for 1 h at RT. Cells were stained in 4,6‐diamino‐2‐phenylindole (DAPI) (Cell Signaling Technology, USA) for 10 min in light protection condition at RT. A fluorescence microscope (#LSM980, Zeiss, Germany) were applied for images capturing.

### 2.9 SA‐β‐Gal Staining

On account of estimating the degree of ageing, we used Senescence β‐Galactosidase Staining Kit (Beyotime, Shanghai, China). Firstly, after exposed to Doxorubicin for 48 h, NFs were washed twice with PBS and β‐galactosidase staining fixative was applied to fix the cells for 15 min. Then, after washing away any reagent with PBS, NFs were stained with staining working solution at 37°C without CO₂ overnight, ensuring the reagent was utilised immediately after it was prepared. Under a phase‐contrast microscopy (CKX53, Olympus Corporation, Japan), cells were observed and captured in random.

### 2.10 Cell Proliferation Analysis

Cell line SNU638 and AGS were seeded respectively into 96‐well plates at a density 5000 cells per well in 200 μL medium and were cultured in 10% serum‐containing medium at 37°C and 5% CO₂. Then, the cells were incubated with CCK‐8 reagent (Dojindo, Japan) and medium for 2 h at 37°C and 5% CO₂. The CCK8 assay was performed at 0, 24, 48 and 72 h. The optical density at 450 nm were measured by means of a microplate reader (BioTek, USA). We performed this assay at three times to obtain average data.

### 2.11 Cell Migration and Wound Healing Assay

Invasion capacity of GC cells was estimated via Transwell Invasion Assay, utilising the 8 μm pore‐size transwell chambers of 24‐well format transwell plates (Corning, USA). After NFs were cultured in 700 μL serum‐containing medium in the lower chambers, they were treated with Doxo (100 nM) for 48 h at 37°C and 5% CO₂. Then, cell lines SNU638 and AGS were resuspended in 200 μL serum‐free medium at a density of 4 × 10^4^ per upper chamber, co‐culturing with senescent NFs. After that, the GC cells still above the membrane of transwell chambers were erased by cotton swabs. 0.5% crystal violet was applied to stain the rest of the GC cells travelling through the membrane for 15 min. Via a phase‐contrast microscopy (#BX51, Olympus Corporation, Japan), cell migration was observed and captured stochastically.

Resuspended in 200 μL serum‐free medium, cells SNU638 and AGS were seeded in culture inserts (IBIDI, Martinsried, Germany) in the 24‐well plates, which were removed when cells were completely adherent after overnight culture at 37°C and 5% CO₂. Then, cells were co‐cultured with senescent NFs with or without downregulation of LINC01579 seeded in transwell chamber with 0.4‐μm pore filter inserts. Images of cell migration after 0, 24 and 48 h were obtained using the phase‐contrast microscopy. ImageJ software was utilised to assess cellular migration rate.

### 2.12 RNA Isolation and Real‐Time Quantitative Polymerase Chain Reaction (RT‐qPCR)

RNAex Pro Reagent (#AG21102, Accurate Biology, Hunan, China) were used to get the total RNA from cells and tissues. The concentration of total RNA was measured, based on which total RNA was reverse‐transcribed to cDNA using Evo M‐MLV PT Primix for qPCR (Accurate Biology, Hunan, China). On a QuantStudio5 Real‐time PCR detection system (#A28574, Applied Biosystems, Piscataway, NJ, USA), qPCR was performed with Evo M‐MLV RT Primix for qPCR (#AG11706, Accurate Biology, Hunan, China). GAPDH was chosen as the internal control. And the primers of targeted genes are listed in Table S2.

### 2.13 In Situ Hybridization (ISH)

ISH was performed using the ISH Kit (Boster Bio‐Engineering Company, Wuhan, China) following the manufacturer's instruction. Two pathologists independently scored slides by microscopy based on both the intensity and proportion of LINC01579–positive cells. The ISH scoring system is as described in the reference article [24].

### 2.14 Tissue Microarray

The GC tissue microarray (TMA, Cat. HStmA180Su19) was obtained from Outdo BioTech (Shanghai, China). The HStmA180Su19 TMA contained 94 GC tissues and the tissues‐associated IHC data of PD‐L1 and PD1. Detailed clinicopathological characteristics of the cohorts were also provided by Outdo BioTech (Shanghai, China). Ethical approval for the study of the TMAs was granted by the Clinical Research Ethics Committee, Outdo BioTech (Shanghai, China).

## 3 Results

### 3.1 Identification of Cellular Senescence‐Related lncRNAs

**FIGURE 2:**
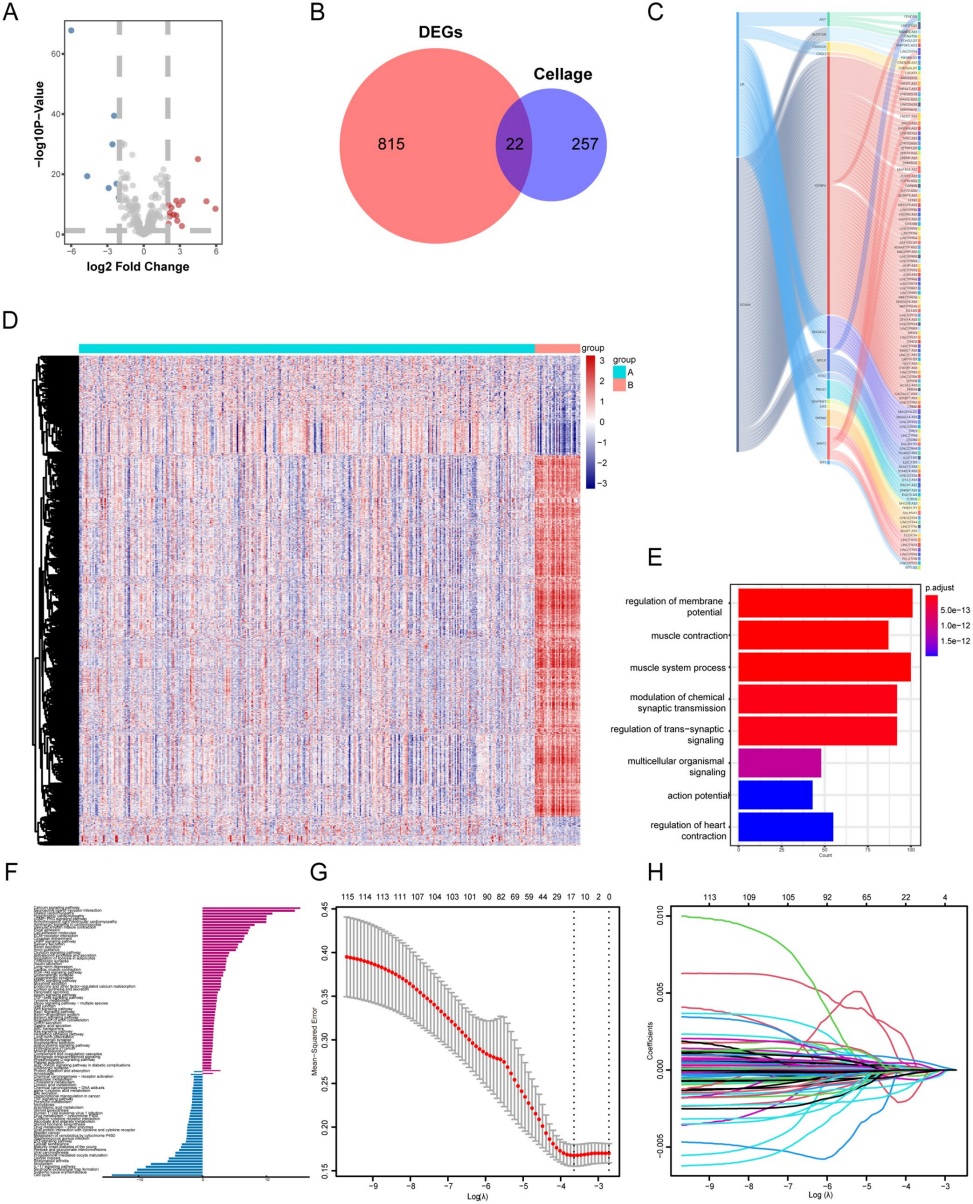
Identification of DEGs and development of cellular senescence‐related lncRNAs signature (A) Volcano plot showed the 22 DEGs identified from 375 STAD patients. (B) Venn plot exhibited the cross genes between DEGs in STAD and genes in CellAge. (C) Sankey diagram demonstrated the relationship between senescence‐related lncRNAs and mRNA of DEGs with different expression levels. (D) Unsupervised hierarchical clustering analysis clustered into two distinct groups: Senescence relevant group and control group, based on expression of senescence‐related lncRNAs. (E) GO functional enrichment analysis. (F) KEGG pathway analysis showed the main related signalling pathways of lncRNAs. (G) Coefficient spectrum of senescence‐related lncRNAs. (H) LASSO correlation coefficients of 16 senescence‐related lncRNAs.

DEGs between GC tissues and normal adjacent tissues were clarified using the RNA expression data from 375 STAD patients (Figure 2A, Table S3). Subsequently, a list of cellular senescence‐related genes was obtained from the CellAge database (Table S4). By comparing the DEGs with list of senescent genes, a total of 22 cellular senescence‐related genes were identified (Figure 2B, Table S5). The mRNA of these genes was considered as cellular senescence‐related RNAs, based on which we identified 116 cellular senescence‐related lncRNAs using Spearman analysis (Figure 2C, Table S6).

### 3.2 Development of a Cellular Senescence Related lncRNA Signature for Outcome Prediction

Cellular senescence is crucial in the carcinogenesis and progression of GC [25]. Given its complexity and variability in specific patient, an accurate prognostic predictive model should be constructed for senescent quantification and improvement of understanding.

After excluding normal tissues, a total of 375 STAD samples from the TCGA dataset were included in the analysis. Using the 116 cellular senescence‐related lncRNAs, unsupervised hierarchical clustering analysis was performed on the 375 STAD samples. As described in the previous method 2.3 and 2.4, the STAD samples were classified into two distinct groups: group A (the control group) and group B (the senescence‐relevant group) (Figure 2D, Table S7). To explore the potential biological functions of senescence, GO and KEGG pathway analyses were conducted in terms of these groups (Figure 2E,F), indicating most of enriched biological processes concentrating on cell cycle, cell signalling processes and senescence program.

The cellular senescence‐related lncRNAs was subjected to LASSO regression analysis to develop a comprehensive and systematic cellular senescence‐related signature. Subsequently, a multi‐gene signature consisting of 16 cellular senescence‐related lncRNAs was constructed (Figure 2G,H). Then, a multivariate Cox regression analysis (*p* < 0.05) was conducted to investigate the predictive function of the cellular senescence‐related signature based on patient outcomes. Subsequently, the long intergenic non‐coding RNA LINC01579 and LINC02306 were identified as independent prognostic cellular senescence‐related lncRNAs for GC, and their expression level are demonstrated as follows: Senescence score = (0.0348 × expression level of LINC01579) + (0.1215 × expression level of LINC02326) (Table S8). Based on the results, the risk score of each patient was calculated in the filtered STAD samples, quantifying the senescence status of individual patients. Using the median senescence score (0.19458) as a threshold, we classified the patients into different prognostic groups, the high‐risk group with equal or higher scores than the threshold, and the other group named low‐risk group.

### 3.3 Estimation and Validation of Cellular Senescence‐Related LncRNAs Signature

**FIGURE 3:**
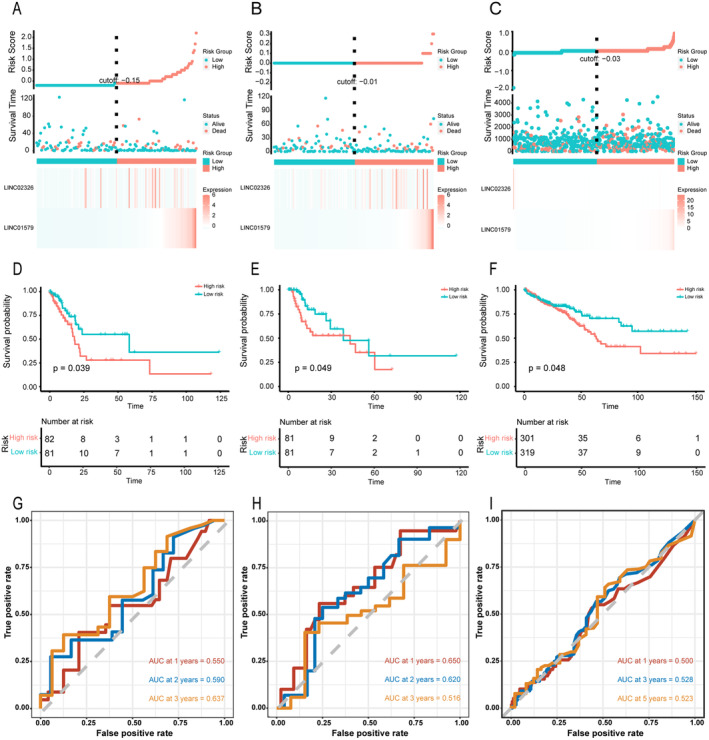
Estimation and validation of senescence‐related lncRNAs signature (A–C) The risk score distribution, Survival status scatter plots and the expression profile of independent prognostic factor of STAD patients in training cohort (A), testing cohort (B) and external cohort (C). (D–F) Kaplan–Meier survival curves of overall survival of high‐risk and low‐risk groups in the training cohort (D), testing cohort (E) and external cohort (F). (G, H) 1‐, 2‐, 3‐year ROC curve of risk scores between high‐risk group and low‐risk group in training cohort (G), testing cohort (H). (I) 1‐, 3‐, 5‐year ROC curve of risk scores between high‐risk group and low‐risk group in external cohort.

The TCGA‐STAD cohort was randomly divided into two sets: the training set and the testing set (Table 1). Figure 3A illustrates the risk scores, survival status and a gene expression heatmap, highlighting the elevated expression of LINC01579 and LINC02306 in high‐risk group. Additionally, Kaplan–Meier analysis (Figure 3D) revealed poor prognosis in high‐risk groups (*p* = 0.039), underscoring the prognostic significance of the cellular senescence‐related lncRNA signature in GC. The time‐dependent ROC analysis demonstrated an AUC of 0.637 at 3 years (Figure 3G).

**TABLE 1:** Clinical and pathological characteristics of STAD patients.

Covariates	Training cohort (*n* = 163)	Testing cohort (*n* = 162)	TCGA‐STAD cohort (*n* = 325)	*p*
Age, no (%)				
Young (< 65)	77 (47.2%)	64 (39.5%)	141 (43.4%)	0.997
Old (≥ 65)	85 (52.2%)	96 (59.3%)	181 (55.7%)	
Unkonwn	1 (0.6%)	2 (1.2%)	3 (0.9%)	
Gender, no (%)				
Female	56 (34.4%)	55 (34.0%)	111 (34.2%)	0.997
Male	107 (65.6%)	107 (66.0%)	214 (65.8%)	
Stage, no (%)				
Stage I	27 (16.6%)	17 (10.5%)	44 (13.5%)	0.104
Stage II	50 (30.7%)	49 (30.2%)	99 (30.5%)	
Stage III	66 (40.5%)	74 (45.7%)	140 (43.1%)	
Stage IV	20 (12.3%)	13 (8.0%)	33 (10.2%)	
Unkonwn		9 (5.6%)	9 (2.8%)	
Group, no (%)				
High risk	84 (51.5%)	76 (46.9%)	165 (50.8%)	0.707
Low risk	79 (48.5%)	86 (53.1%)	160 (49.2%)	

The efficiency of cellular senescence‐related lncRNA signature was assessed in both the testing set and the TCGA set. In the two sets, LINC01579 and LINC02326 are highly enriched in high‐risk group (Figure 3B, Figure S1A). The Kaplan–Meier curve showed significantly better survival outcomes in the low‐risk group in the testing cohort (*p* = 0.049), and same as that of the TCGA‐STAD cohort (*p* = 0.0026) (Figure 3E, Figure S1B). The time‐dependent ROC analysis, using the training set model, indicated an AUC of 0.650 at 1 year and 0.637 at 3 years in testing cohort (Figure 3H), confirming the signature's strong predictive efficacy. Same tendency can be observed in TCGA‐STAD cohort (Figure S1C).

To further validate the robustness of the risk scores, external validation was conducted using the TCGA‐COAD and TCGA‐READ cohorts, both of which represent digestive carcinomas, making them suitable for this analysis. Using the same formula, risk scores for samples in the TCGA‐COAD and TCGA‐READ cohorts were calculated, allowing the cohorts to be stratified into high‐risk and low‐risk groups. The distribution of risk scores, survival status and a heatmap of gene expression are presented in Figure 3C. As shown in Figure 3F, the low‐risk group exhibited better survival outcomes (*p* = 0.048). The area under the curve (AUC) values for 1‐, 3‐ and 5‐year survival predictions were 0.500, 0.528 and 0.523, respectively (Figure 3I). In conclusion, our cellular senescence‐related lncRNA signature has demonstrated accuracy and effectiveness in predicting the OS of GC patients, holding promise as a prognostic tool offering valuable insights into patient outcomes. Incorporating specific cellular senescence‐related lncRNAs aids in understanding and management of this disease.

### 3.4 Cellular Senescence Related LncRNAs Signature in Diverse Clinical Subgroups

**FIGURE 4:**
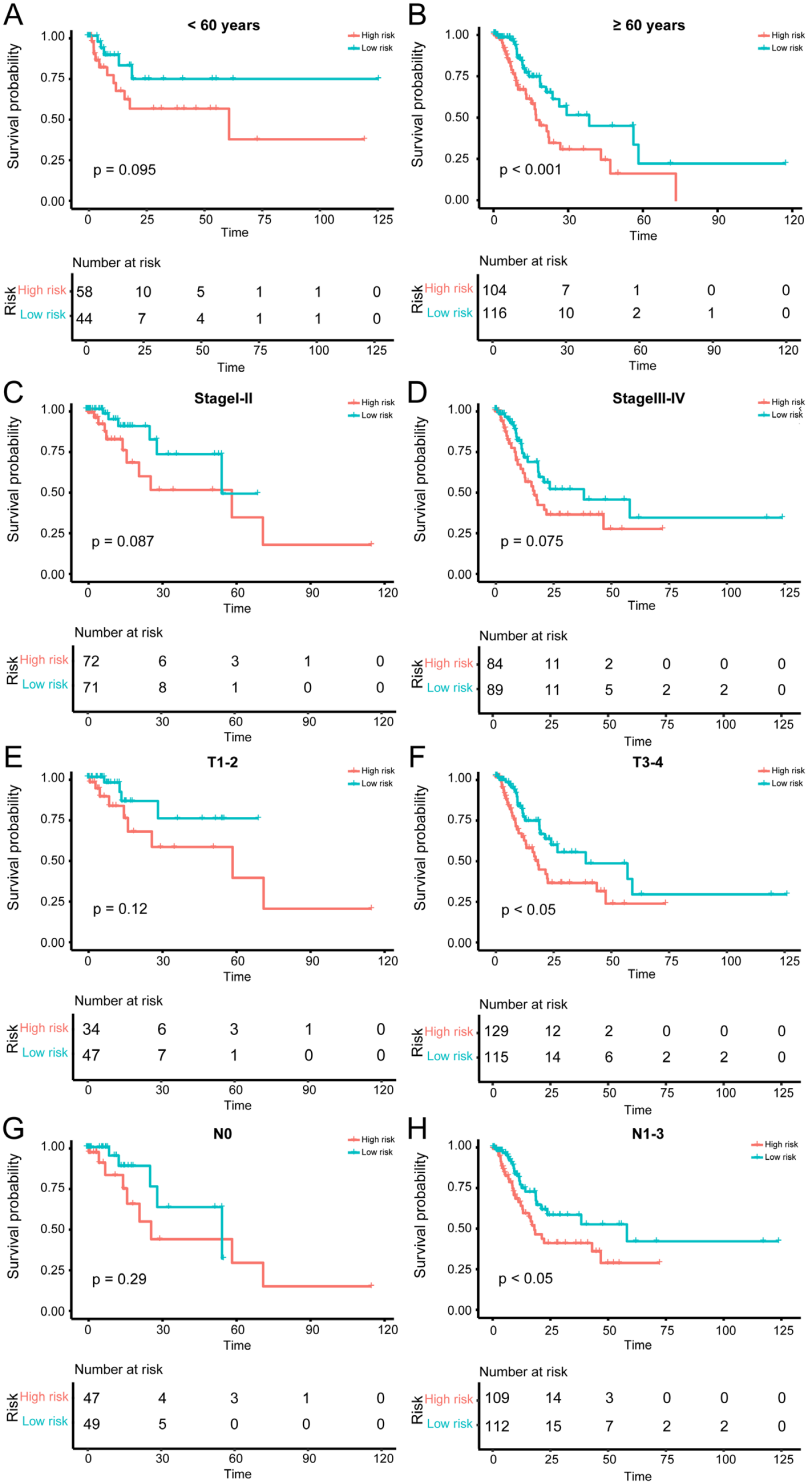
OS analysis of senescence related lncRNAs signature in diverse clinical subgroups Kaplan–Meier estimates of OS based on the senescence‐related lncRNAs signature in clinical subgroups stratified ground on age (A, B), stage (C, D), T (E, F), N (G, H) in the TCGA‐STAD cohort.

To examine the predictive potential of senescence‐related lncRNAs signature in different clinical subgroups, patients were divided into diverse subgroups on the basis of age, TNM stage, T stage, N stage using Kaplan–Meier analysis of the OS in different subgroups. Shown in Figure 4A–H, the results demonstrated that in TCGA‐STAD cohort, patients in low‐risk group had better OS versus patients in high‐risk group in < 60 (*p* = 0.095), ≥ 60 (*p* < 0.001), stage I–II (*p* = 0.087), stage III–IV (*p* = 0.075), T1‐2 (*p* = 0.12), T3‐4 (*p* < 0.05), N0 (*p* = 0.29), N1‐3 (*p* < 0.05). The results indicated that our senescence‐related lncRNAs signature performed better in subgroups of age ≥ 60, T3‐4 and N1‐3 that were typically regarded as high‐risk people and advanced tumour stage with less 5 years OS and higher mortality [26].

### 3.5 Mutation Status of the TCGA‐STAD Cohort

**FIGURE 5:**
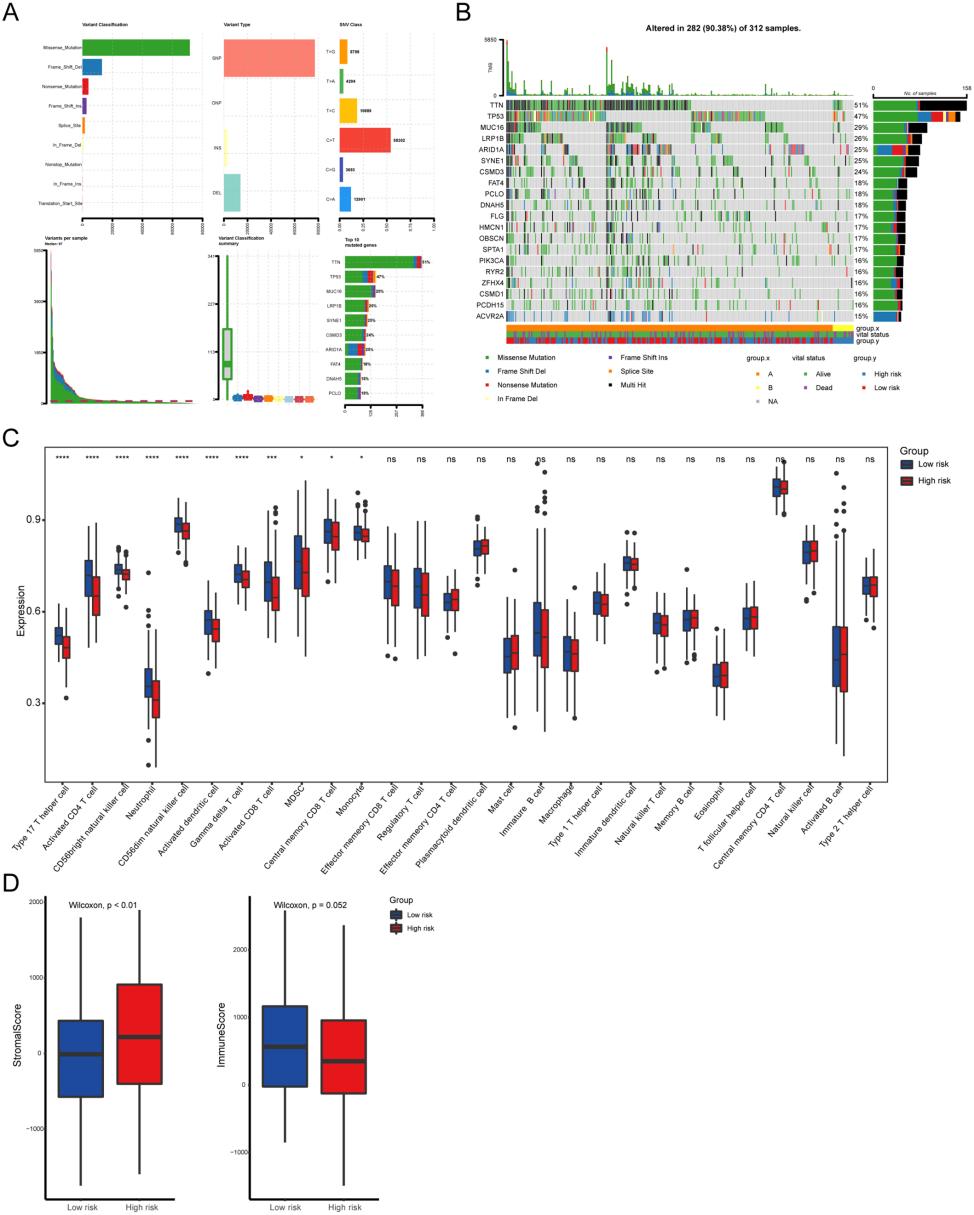
Assessment of the tumour microenvironment and immune cell infiltration (A) Summary plot of mutation information showed the mutation landscape of STAD, including mutative classification, types and classes. (B) Waterfall chart exhibited the top mutative genes of high‐risk and low‐risk group in STAD. (C) Immune infiltration differences between high‐ and low‐risk groups in STAD. (D) Comparison of stromal score and immune score between high‐ and low‐risk groups. *: *p* < 0.05, **: *p* < 0.01, ***: *p* < 0.001.

In the context of the mutation data from TCGA, we conducted a comprehensive analysis using a summary plot of mutation information and a waterfall plot to investigate the intricate relationship between gene mutation levels and prognostic predictive signatures (Figure 5A,B). The summary plot of mutation information highlighted that the analysed mutations can be primarily classified based on their mutative influence, with a predominant presence of missense mutations, approximately four times more prevalent than frame shift deletion mutations, which ranked secondarily (Figure 5A). The primary type of mutation variants observed were single nucleotide polymorphisms (SNPs) or single nucleotide variations (SNVs), predominantly characterised by C to T mutations. The top mutative genes identified are depicted in Figure 5A, while the waterfall plot in Figure 5B provides further elucidation. Notably, the plot demonstrated that 51% of patients exhibited TTN mutations (Figure 5B).

Existing studies have reported an association between TTN mutations and increased tumour mutation burden, as well as their role as enhancers of immune checkpoint blockage, thereby accelerating the constructive processes and promoting the accumulation of immunosuppression within the TME [27, 28]. Additionally, the TCGA‐STAD cohort exhibited a 47% incidence of TP53 mutations, ranking as the second most prevalent mutation among the top 10 mutative genes (Figure 5B). TP53 is widely recognised as a crucial regulator in cellular cycle arrest and senescence initiation programs [29]. Hence, it is imperative to further explore the potential connections between TTN mutations, which are relevant factors in the tumour immune microenvironment, and TP53, the initiator of cellular senescence. These factors play significantly pivotal roles in the process of GC, warranting a comprehensive elucidation of their detailed interplay with senescence‐related lncRNAs [28, 30].

### 3.6 Assessment of the Tumour Microenvironment and Immune Cell Infiltration

To elucidate the intricate relationship between senescence‐related lncRNAs and tumour immune responses, a comprehensive evaluation of immune cell infiltration in the TME was conducted, aiming to assess the abundance of distinct immune cell populations in both high‐ and low‐risk groups (Figure 5C). As depicted in the plot, the high‐risk group exhibited significantly lower levels of immune cell infiltration across various cell types compared to the low‐risk group. Particularly noteworthy was the decreased infiltration of active CD4+ and CD8+ T cells (key effectors of specific immunity) and Th17 cells (a significant subset of T helper cells) in the high‐risk group. Moreover, immune cells involved in nonspecific immunity, such as neutrophils, monocytes and natural killer cells, also displayed diminished infiltration in the high‐risk group. Interestingly, the infiltration level of myeloid‐derived suppressor cells (MDSCs) was observed to be decreased in the high‐risk group.

We hypothesise that this phenomenon can be attributed to the overall compromised immune response resulting from severe immunosuppression within the high‐risk group, despite the concurrent decrease in the abundance of immunosuppressive cells. The assessment of TME components, including stromal score and immune score, further aligns with the diminished immune cell infiltration observed in Figure 5C,D. Specifically, the high‐risk group exhibited elevated stromal scores and decreased immune scores in comparison to the low‐risk group, indicating an essential state of immunosuppression within the high‐risk group.

Collectively, these findings consistently support the notion that the high‐risk group exhibits an immunosuppressive microenvironment that may contribute to an unfavourable prognosis.

### 3.7 Co‐Culturing With Senescent NFs Affected Cellular Proliferation and Migration Capacity of GC Cells

The results of the risk score analysis demonstrate that LINC01579 serves as an independent prognostic factor, exhibiting significant upregulation in the high‐risk groups of the training, testing, TCGA‐STAD and TCGA COAD+READ cohorts and enrichment compared with LINC02326 (Figure 3A–C, Figure S1A). This finding strongly suggests a close association between elevated LINC01579 expression and an unfavourable prognosis in GC. However, the precise role of LINC01579 in the initiation and progression of GC remains largely unexplored, necessitating further investigation.

**FIGURE 6:**
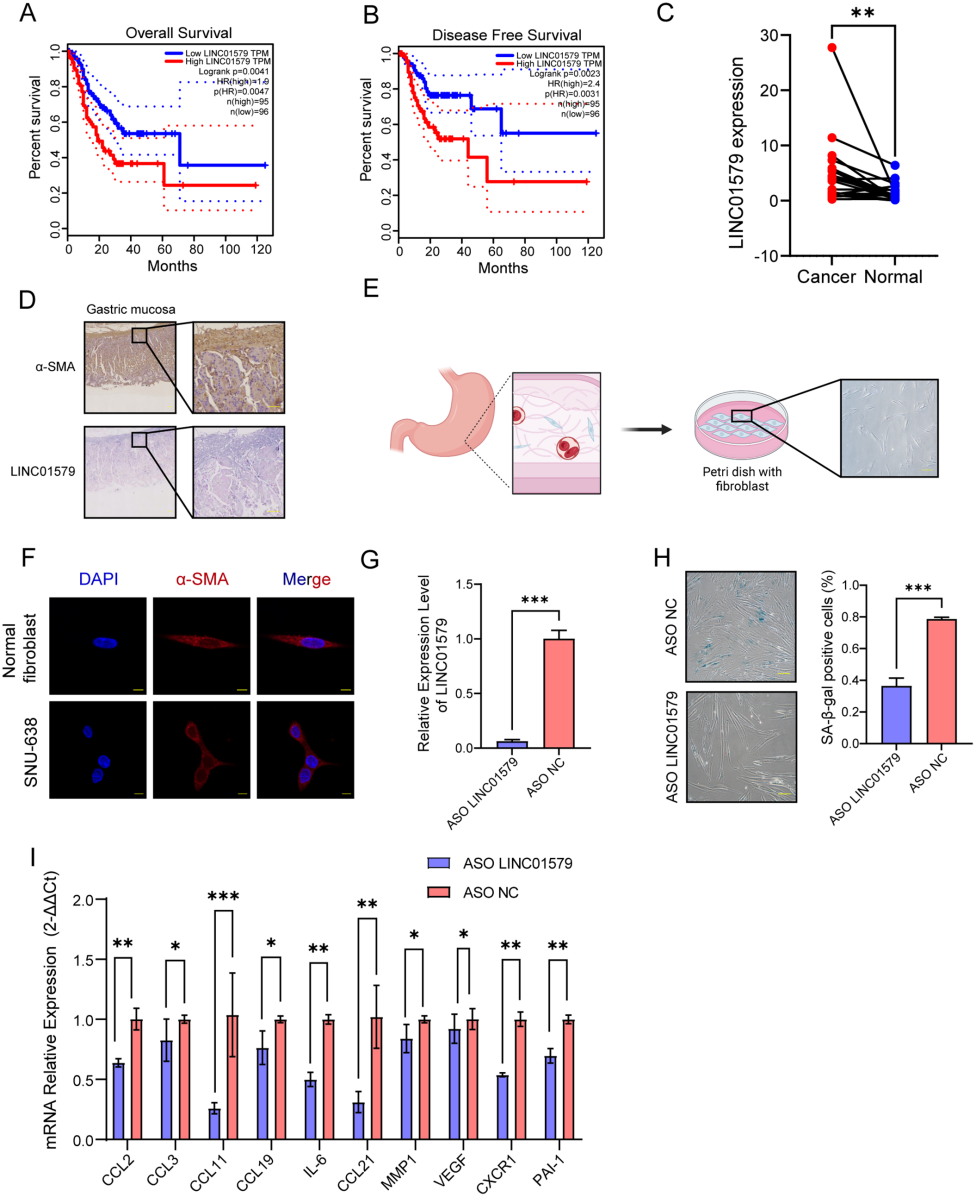
The knockdown of LINC01579 inhibits cellular senescence of NFs in vitro (A) Overall survival of high‐ and low‐expression of LINC01579 group of TCGA‐STAD cohort in GEPIA. (B) Disease free survival of high‐ and low‐ expression of LINC01579 group of TCGA‐STAD cohort in GEPIA. (C) qPCR analysis of LINC01579 in GC and adjacent normal tissues (*n* = 22, normalised to GAPDH). (D) ISH showed the spatial localization of LINC01579 within the organisation. (E) Schematic of the origin of NFs and its image under microscope. (F) Immunofluorescence analysis verified the fibroblastic phenotype of cell line NFs. (G) Verification of knock down of LINC01579 in NFs using qRT‐PCR. (H) SA‐β‐gal staining exhibited the different senescent phenotype of NFs with or without LINC01579 downregulation. (I) qRT‐PCR showing the difference of SASP expression in NFs with or without LINC01579 downregulation. *: *p* < 0.05, **: *p* < 0.01, ***: *p* < 0.001.

To shed light on the potential involvement of LINC01579 in the malignant progression of GC, we directed our attention towards the TME, as senescence has been shown to influence TME and contribute to tumour progression [11, 12, 13]. Fibroblasts were regarded as cells that particularly susceptible to the senescence‐related influence [11]. Consequently, we sought to examine the impact of LINC01579, a senescence‐related lncRNA, on fibroblasts concurrently. Figure 6A,B demonstrated the effect of LINC01579 expression level on OS and disease‐free survival of TCGA‐STAD cohort, indicating GC patients with low LINC01579 expression have better survival. The qPCR analysis of LINC01579 in GC and adjacent normal tissues was performed to evaluate the tissue expression difference, showing the higher expression of LINC01579 in GC tissues (Figure 6C). The cellular location of LINC01579 was identified using ISH, depicting an elevated expression of LINC01579 in interstitium compared to glandular tissue of adjacent tissues (Figure 6D).

We performed a tissue microarray to further evaluate the difference between high‐ and low‐ LINC01579 expression in GC. As Figure S1A shown, ISH was used to assess LINC01579 expression levels, categorising samples into high‐ and low‐expression groups. Notably, a higher PD‐L1 expression level was observed in the high LINC01579 expression group (Figure S2B), compared to the low LINC01579 expression group. The expression levels of PD1 showed no significant difference between the high and low LINC01579 expression groups (Figure S2C). It suggests that the elevated LINC01579 expression might correlate with the PD‐L1 expression by influencing the cellular senescence [31, 32], indicating that the high LINC01579 expression in the tumour may associate with local immunosuppression, thereby influencing tumour progression and prognosis from an immunological perspective. Additionally, Table S9 reveals a correlation between the age factor and LINC01579 expression level, with samples from patients older than 55 years old showing higher LINC01579 expression. This finding provides additional evidence that aging is closely associated with the expression of LINC01579 in GC. This finding aligns well with our proposed model, suggesting that senescence‐related mechanisms may significantly regulate LINC01579 expression and contribute to the progression and pathogenesis of GC.

In this study, we selected NFs derived from GC tissues rather than cancer‐associated fibroblasts as our experimental model in condition to the purpose to estimate the effect of LINC01579 on prognosis (Figure 6E). To ensure their identity as fibroblasts, the expression of α‐smooth muscle actin (α‐SMA) was confirmed, a well‐established marker of fibroblast (Figure 6F). To investigate the functional role of LINC01579, we employed an ASO designed to specifically target and downregulate LINC01579 expression. The efficiency of LINC01579 interference was evaluated using qRT‐PCR (Figure 6G).

**FIGURE 7:**
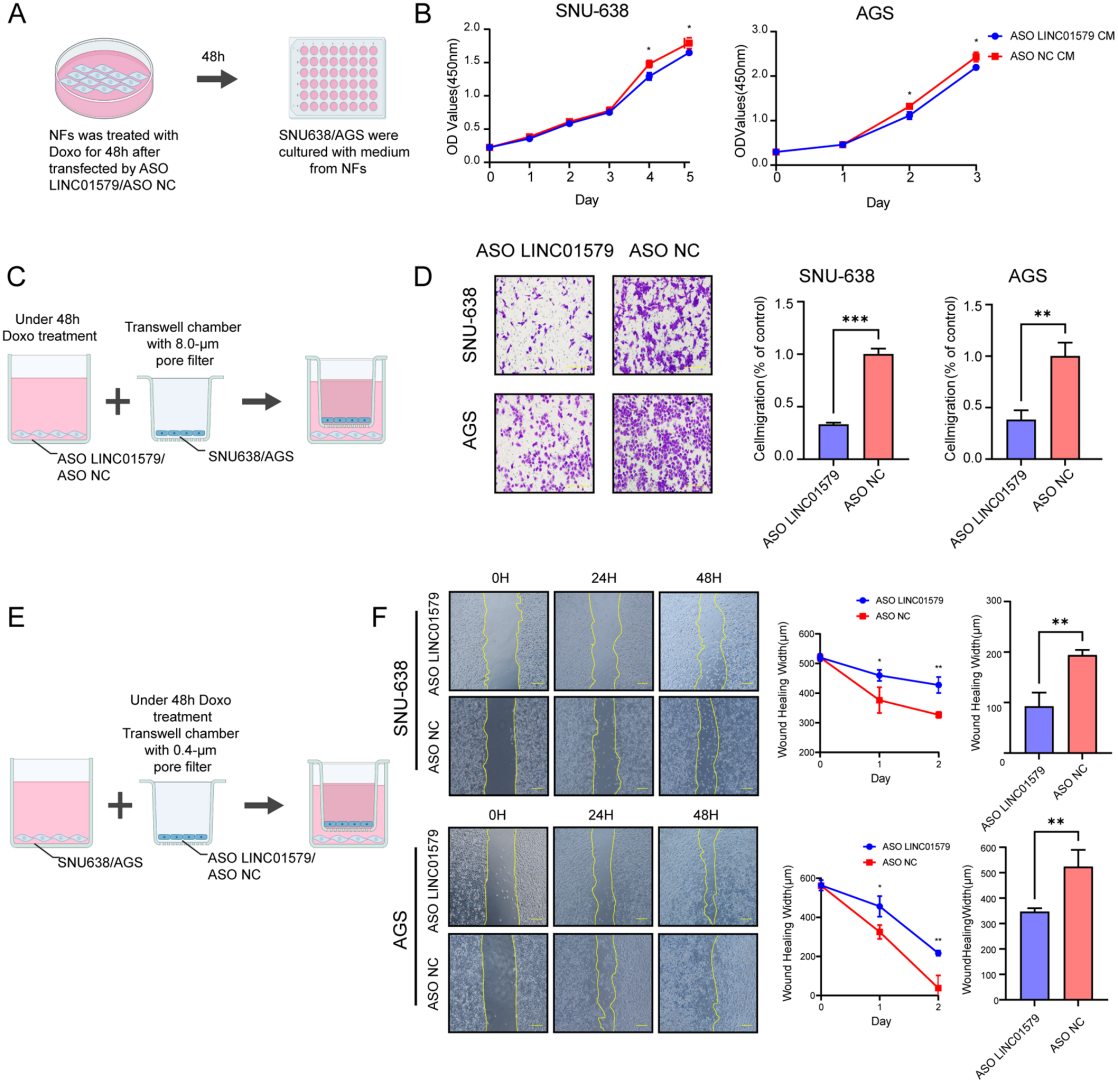
The knockdown of LINC01579 in NFs promotes the proliferation and migration of GC cells (A) Schematic of CCK‐8 assay. (B) CCK‐8 assay determining the proliferation of GC cell lines cultured with medium from NFs with or without LINC01579 downregulation. (C, E) Schematic of transwell migration assay and wound‐healing assay. (D, F) Transwell migration assay (D) and wound‐healing assay (F) exhibiting the migratory difference of GC cells co‐cultured with NFs with or without LINC01579 downregulation. *: *p* < 0.05, **: *p* < 0.01, ***: *p* < 0.001.

Subsequently, we assessed cellular senescence by employing the senescence‐associated β‐galactosidase (SA‐β‐gal) staining method (Figure 6H). Our findings, as depicted in Figure 6G, reveal a pronounced increase in senescence levels in NFs treated with LINC01579 ASO compared to the control group following a 48‐h chemotherapeutic treatment. The quantification of SASP in NFs following a 48‐h chemotherapeutic intervention was assessed via qRT‐PCR analysis (Figure 6I). Moreover, we evaluated the impact of senescent NFs with downregulated LINC01579 on the proliferation and migration capacity of GC cells. Proliferation assays, specifically the CCK‐8 assay, demonstrated a significant enhancement in GC cell proliferation when cultured with the medium from senescent NFs compared to controls (Figure 7A,B). To further investigate the migratory potential of GC cells, we performed transwell migration and wound‐healing assays. As illustrated in Figure 7C–F, GC cells co‐cultured with senescent NFs exhibiting downregulated LINC01579 displayed a markedly decreased migratory ability compared to those co‐cultured with senescent NFs expressing normal levels of LINC01579.

In summary, our results suggest that downregulation of LINC01579 in NFs limits the proliferation and migration of GC cells. These findings provide valuable insights into the potential role of LINC01579 in GC progression, highlighting the need for further investigations to comprehensively elucidate its underlying mechanisms and clinical implications.

## 4 Discussion

A further exploration of TME and an effective tool to evaluate its comprehensive signature are of great significance and imminently needed for prognostication of GC and for performing more advanced therapy strategies in clinical settings. In this study, we identified 116 cellular senescence‐related lncRNAs following a screening of senescence‐associated genes. Based on these findings, we constructed a prognostic model that focused on two independent prognostic factors: LINC01579 and LINC02326. The efficacy of this model, namely the cellular senescence‐related lncRNA signature, was evaluated across the testing, training, TCGA‐STAD cohorts, and combined TCGA‐COAD and READ cohorts, revealing that patients with elevated levels of LINC01579 experienced significantly lower OS. Subsequently, we assessed the predictive potential of the model within various clinical subgroups categorised by age, TNM stage, T stage and N stage. Furthermore, we analysed the mutation status of the TCGA‐STAD cohort, finding that 51% of patients harboured TTN mutations. An evaluation of the TME and immune cell infiltration indicated that patients with high LINC01579 expression exhibited an immunosuppressive microenvironment, which may contribute to a poorer prognosis. Finally, vitro study have proved that the high level of LINC01579 results in elevated cellular proliferation and migration of GC cells, probably by facilitating more SASP production.

Senescence is an irreversible arrest of cellular proliferation, featuring an activation of a SASP [33]. Simultaneously, it is described as a stress response that can be triggered by chemotherapy [34]. Although the importance of cellular senescence and SASP phenotype in malignant progression has been acknowledged, fundamental principles on how cellular senescence influences the progression and the prognosis of GC especially, remain obscure. Recent studies have stated that the impact of stromal cellular senescence levels for prognosis in diverse malignancies cannot be underestimated. As an example, senescence can be induced as a profound secretory alteration in fibroblasts, a predominant stromal constituent and ECM remodeler, in response to chemotherapy [7, 34, 35, 36]. In our studies, fibroblasts were observed to express senescent marker Sa‐β‐gal in response to exogenous stress qualified by staining. And senescent fibroblasts might play as a vital stromal component and SASP provider to explore GC cells' behaviour and individual prognosis.

Due to the complex and considerable regulatory network within and among GC cells, it is rather arduous to elucidate underlying mechanisms in aspect of GC's initiation and development. With further studying of impact of lncRNAs on gastric malignancy, we recognised its crucial impact on malignant regulative program. Then we desire to know if lncRNAs play a pivotal role in cellular senescence in GC progression. In our study, we determined senescence‐related genes, subsequently, gene IGFBP6 was founded and its downstream lncRNAs were uncovered, one of which is LINC01579 (Table S3). Considering LINC01579 is one independent prognostic factor in GC, we hold a belief that IGFBP6 potentially acts as a key molecule in the underlying regulatory network. As our expected, IGFBP6 participates in regulation of cellular proliferation and can be upregulated by oncogenes in GC, regarded as one of biomarkers of GC [37, 38]. In Shen’ work, it is determined as one of hub genes in senescence‐related pathway in GC, consistent with our conclusion [39]. Consequently, it is reasonable to hypothesise that lncRNAs downstream of IGFBP6 would have a crucial role in cell proliferation and regulatory mechanism in senescent process.

Uncovering senescence‐related lncRNAs was on the basis of the upstreaming mRNAs. In our study, LINC01579 was chosen as an independent prognostic factor of senescence in GC. Several studies have shown its various functions in different research areas. The functional role of LINC01579 in glioblastoma (GBM) has been elucidated in a recent study. It has been proposed that LINC01579 acts as a competing endogenous RNA (ceRNA) by interacting with miR‐139‐5p, thereby modulating the expression of EIF4G2 [40]. The downregulation of LINC01579 in GBM has been associated with suppressed cellular proliferation and enhanced cellular apoptosis [15, 40]. In our investigation, we sought to examine the impact of LINC01579 on tumour biological behaviour in GC and elucidate its precise impacts on the senescence process in stromal cells. Not as a ceRNA, in Yang Chai's work, in the present work, LINC01579 was viewed as senescence promotor and SASP regulator in stroma, which was silenced in fibroblasts of TME in our further exploration [40]. As our expected, silencing LINC01579 significantly attenuated the senescence phenotype of and stromal SASP factors, compared to the control group under treatment of chemotherapeutic agents. In addition, LINC01579 has been identified as an angiogenesis‐related lncRNA that holds predictive value for the prognosis of GC [41]. LINC01579 was knocked down in AGS and MKN45 cells by siRNA, whose inhibition of the proliferation was observed. The decreased ability of cellular proliferation is consistent with our results.

Our model examines the relationship between aging and survival in GC patients. In the TCGA‐STAD cohort of 375 patients, those aged 65 and older make up over half of the population. Additionally, male patients and those classified as Stage III‐IV in the TNM system also represent more than half of the cohort. Thus, our model primarily targets a high‐risk population. In subsequent evaluations of the model's predictive efficiency across different clinical subgroups, it demonstrates better performance in subgroups of high‐risk individuals and advanced tumour stages. LINC01579 has been reported in uterine corpus endometrial carcinoma, gliomas, glioblastoma and Hirschsprung's disease [40, 42, 43, 44], with only one study related to GC specifically mentioning gastric adenocarcinoma [41]. In three of these similar studies [41, 42, 43], LINC01579 primarily played a participatory role in model construction and was significantly enriched in high‐risk groups, aligning with our findings. Among the published literature on LINC01579, we noted that the primary clinical cohort utilised was TCGA [41, 42, 43], which is consistent with our study. Additionally, these articles consistently emphasise the potential role of LINC01579 in tumour proliferation and metastasis, further validating our hypothesis that LINC01579 promotes tumour invasion and migration. Moving forward, integrating multicenter clinical data and patient populations with diverse ethnic backgrounds will help further confirm the clinical applicability and generalizability of LINC01579. This will provide a solid foundation for developing more effective personalised treatment strategies and offer new insights into tumour biology research.

This research provides several key advantages. Firstly, our model primarily investigates the relationship between cellular senescence and GC prognosis, an area that remains insufficiently explored in the current GC literature. Furthermore, our study integrates lncRNAs to preliminarily explore the effects of senescence‐associated lncRNAs on GC cells, thereby establishing a foundation for future investigations into the yet‐to‐be‐elucidated mechanisms involved. Moreover, in our experimental design, we employed normal human fibroblasts to simulate the stromal components instead of tumour‐associated fibroblasts. This approach ensures a more accurate representation of the post‐operative GC tissue microenvironment and the impact of senescence on GC cells more closely reflects the recurrence scenarios encountered in GC patients. Consequently, this enhances the accuracy and reliability of our experimental findings. In our investigation, we observed that the expression levels of LINC01579 within tissues may significantly impact the immune status of patients. Specifically, the high‐risk group demonstrated a statistically significant reduction in immune cell infiltration during the immune cell infiltration analysis, suggesting that this observation merits further exploration in subsequent studies. The interplay between cellular senescence and immune response may be pivotal in shaping future therapeutic approaches to tumours. A comprehensive examination of senescence could yield critical insights into the management of the SASP induced by chemotherapy, thereby potentially informing strategies to mitigate recurrence associated with such treatments. To clinically translate LINC01579 as an independent prognostic factor in GC, one potential application is its incorporation into practical diagnostic tools aimed at predicting the risk of occurrence and progression of the disease at multiple points. Following a comprehensive exploration of the mechanisms through which LINC01579 functions in cancer, we anticipate that it will play a crucial role in risk stratification models, thereby enhancing clinical decision‐making as the population ages.

There exist several limitations in this study at the same time. First, further validation in multicenter studies involving diverse cohorts or larger patient populations is necessary. Our study relies on data from the TCGA‐STAD cohort, which is because the limitations of the depth and accuracy of sequencing that there exist few databases offering sufficient and good‐quality data on lncRNAs. Additionally, mechanistic insights into LINC01579 as a senescence‐related lncRNA in GC require further in vivo experimental investigations. Last, in vitro experiments are based on 2‐dimensional cell models, which are different from actual tumour tissue and a significant gap exists in the aspects of cellular signal regulation and action.

## 5 Conclusions

In summary, our study successfully identified a promising prognostic model for GC, emphasising the significance of the independent prognostic factor LINC01579. We investigated its role in GC progression, conducted mutation analysis and explored senescence‐immune cell links. Overall, our work has potential clinical applications and sheds light on the relationship between senescence‐related lncRNAs and GC progression and carcinogenesis, providing a foundation for future exploration in this field.

## Author Contributions

**Jiayong He:** data curation (equal), investigation (equal), visualization (equal), writing – original draft (equal). **Ziyi Fu:** data curation (equal), investigation (equal), visualization (equal), writing – original draft (equal). **Boya Zou:** data curation (equal), investigation (equal), visualization (equal), writing – original draft (equal). **Xuetao Lei:** validation (equal), writing – review and editing (equal). **Linhan Lei:** validation (equal), writing – review and editing (equal). **Qingbin Yang:** funding acquisition (equal), supervision (equal), writing – review and editing (equal). **Guoxin Li:** funding acquisition (equal), supervision (equal), writing – review and editing (equal).

## Conflicts of Interest

The authors declare no conflicts of interest.

## Supporting information


**Figure S1.** Validation of senescence‐related lncRNAs signature in the TCGA‐STAD cohort (A) The risk score distribution, Survival status scatter plots and the expression profile of independent prognostic factor of patients in the TCGA‐STAD cohort. (B) Kaplan–Meier survival curves of overall survival of high‐risk and low‐risk groups in TCGA‐STAD cohort. (C) 1‐, 2‐, 3‐year ROC curve of risk scores between high‐risk group and low‐risk group in TCGA‐STAD cohort.
**Figure S2.** Tissue microarray shows the LINC01579 expression level related with immune factor (A) ISH revealed differential expression levels of LINC01579 among the samples. (B) PD‐L1 expression level in different LINC01579 expression groups. (C) PD1 expression level in different LINC01579 expression groups.

Table S1.

Table S2.

Table S3.

Table S4.

Table S5.

Table S6.

Table S7.

Table S8.

Table S9.

## Data Availability

The datasets supporting the conclusions of this article are included within the article and its additional files.
