# Supplementary material for: Identification the Cellular Senescence Associated lncRNA LINC01579 in Gastric Cancer
Source: J Cell Mol Med. 2025 Jan 24;29(2):e70360. doi: 10.1111/jcmm.70360 (PMC11760997; doi:10.1111/jcmm.70360)
Supplement: Supplementary file 1 — Figure S1. Validation of senescence‐related lncRNAs signature in the TCGA‐STAD cohort (A) The risk score distribution, Survival status scatter plots and the expression profile of independent prognostic factor of patients in the TCGA‐STAD cohort. (B) Kaplan–Meier survival curves of overall survival of high‐risk and low‐risk groups in TCGA‐STAD cohort. (C) 1‐, 2‐, 3‐year ROC curve of risk scores between high‐risk group and low‐risk group in TCGA‐STAD cohort. Figure S2. Tissue microarray shows the LINC01579 expression level related with immune factor (A) ISH revealed differential expression levels of LINC01579 among the samples. (B) PD‐L1 expression level in different LINC01579 expression groups. (C) PD1 expression level in different LINC01579 expression groups. [file JCMM-29-e70360-s005.docx]

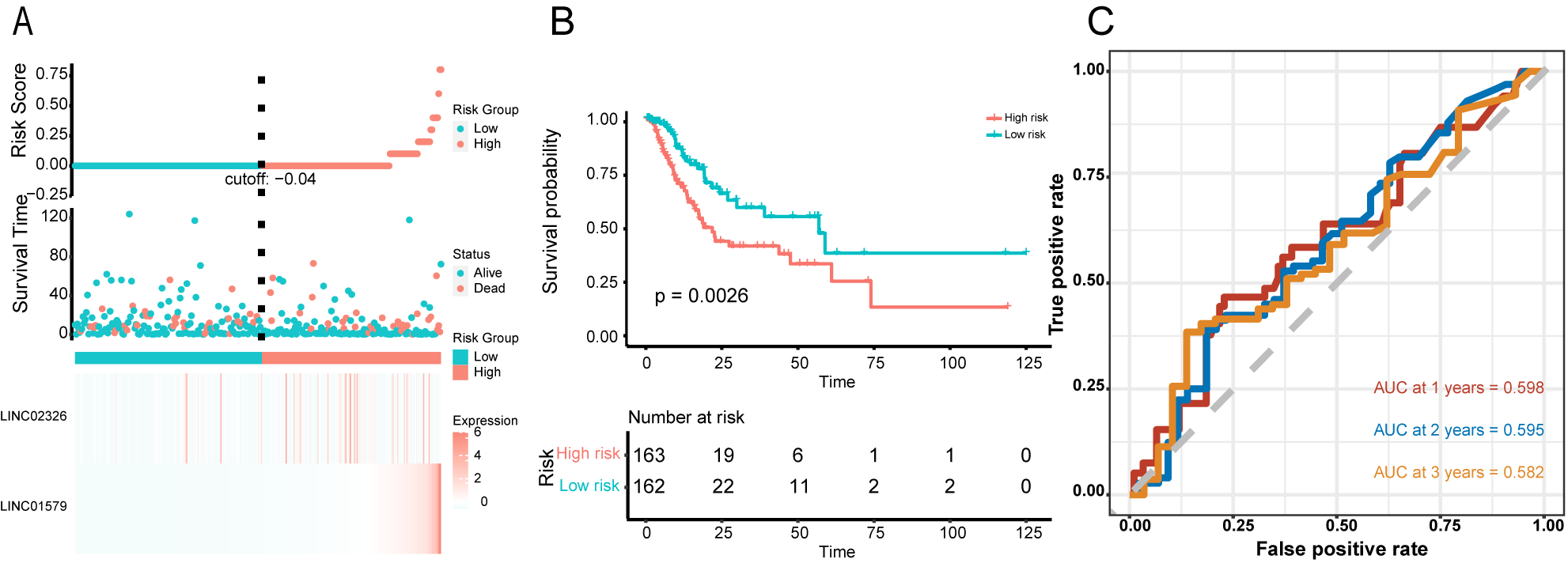


**Supplementary Figure 1** **Validation of senescence-related lncRNAs signature in the TCGA-STAD cohort (A)** The risk score distribution, Survival status scatter plots, and the expression profile of independent prognostic factor of patients in the TCGA-STAD cohort. **(B)** Kaplan-Meier survival curves of overall survival of high-risk and low-risk groups in TCGA-STAD cohort. **(C)** 1-, 2-, 3-year ROC curve of risk scores between high-risk group and low-risk group in TCGA-STAD cohort.


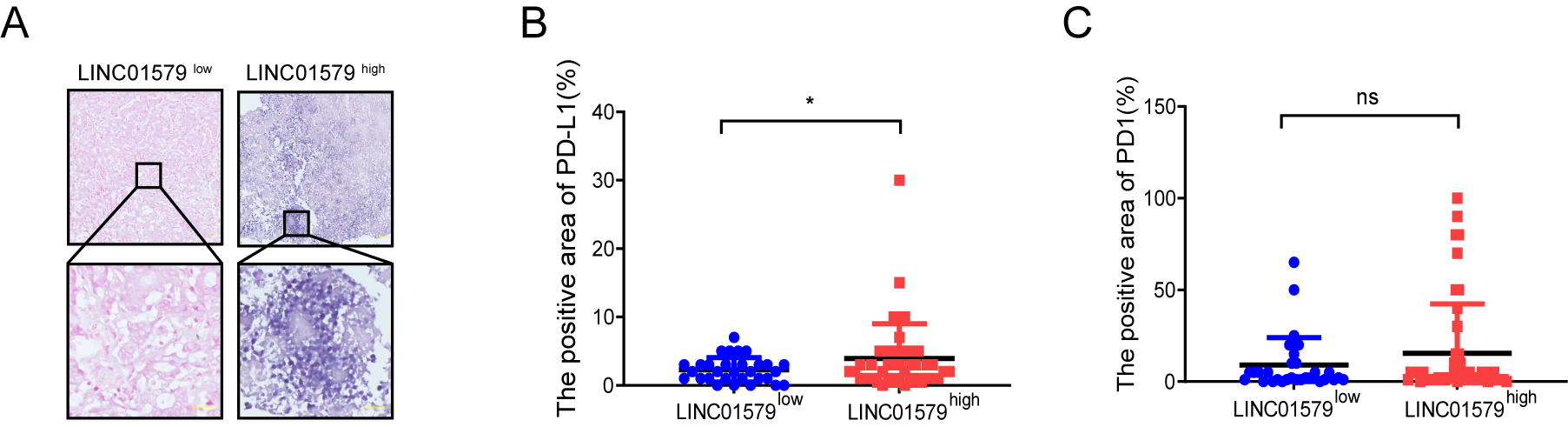


**Supplementary Figure 2** **Tissue microarray shows the LINC01579 expression level related with immune factor (A)** ISH revealed differential expression levels of LINC01579 among the samples. **(B)** PD-L1 expression level in different LINC01579 expression groups. **(C)** PD1 expression level in different LINC01579 expression groups.
